# Control of Vector-Borne Human Parasitic Diseases

**DOI:** 10.1155/2016/1014805

**Published:** 2016-12-20

**Authors:** Fernando A. Genta, Hector M. Diaz-Albiter, Patrícia Salgueiro, Bruno Gomes

**Affiliations:** ^1^Oswaldo Cruz Institute-Oswaldo Cruz Foundation (IOC-FIOCRUZ), Av. Brasil 4365, Manguinhos, 21040-360 Rio de Janeiro, RJ, Brazil; ^2^National Institute of Science and Technology for Molecular Entomology (INCT-EM), Av. Carlos Chagas Filho 373, Centro de Ciências da Saúde, Bloco D-SS, Sala 05, Cidade Universitária, RJ, Brazil; ^3^Wellcome Trust Centre for Molecular Parasitology, University of Glasgow, University Place, Glasgow G12 8TA, UK; ^4^Centro de Investigación y Estudios en Salud Animal, Facultad de Medicina Veterinaria y Zootecnia, Universidad Autónoma del Estado de México, Av. Instituto Literario No. 100 Oriente, Col. Centro., 50000 Toluca, MEX, Mexico; ^5^Unidade de Parasitologia Médica, Global Health & Tropical Medicine, GHTM, Instituto de Higiene e Medicina Tropical, IHMT, Universidade Nova de Lisboa, Rua da Junqueira 100, 1349-008 Lisbon, Portugal; ^6^Department of Vector Biology, Liverpool School of Tropical Medicine, Pembroke Place, Liverpool L3 5QA, UK

Vector-borne diseases (VBD) transmitted by arthropods are responsible for over 1 billion cases and 1 million deaths every year, corresponding to at least 17% of all infectious diseases in human populations [1]. Among them, we can find malaria, leishmaniasis, onchocerciasis, lymphatic filariasis, Chagas disease, and African trypanosomiases, as well as several arboviral diseases (arthropod-borne virus) such as dengue and Zika virus. Some of these have reemerged in new parts of the world and have become a topic of growing importance in public health and in political and scientific agendas [2]. Several factors are contributing towards the reemergence of VBDs. On the one hand, the spread of resistance to drugs in pathogens has become a major obstacle for the effective treatment of some VBDs [3], and the emergence of new strains of arboviruses (*e.g., *Zika virus in Brazil) has created new challenges for health care systems [4]. On the other hand, an increase in insecticide resistance is threatening the sustainability of vector control programmes in several tropical regions [5]. Additionally, the expansion of different vector populations due to climate change is becoming a growing concern in temperate countries, where vector control programs have been discontinuous for almost 50 years [6, 7]. The scientific community has been trying to overcome these challenges by creating new strategies and tools to improve the diagnosis and treatment of VBDs and by developing new methodologies and targets for vector control campaigns. This special issue of BioMed Research International compiles nine topical articles that explore recent advances in research of an eclectic range of pathogens, vectors, and human diseases affecting several regions of the world.

Malaria remains the human parasitic disease with the highest burden and with risk of reemergence in several areas worldwide. In this special issues there are four papers regarding malaria.

Dahalan et al. [8] focus on a promising target for antimalarial drug, the mitogen-activated protein kinase 2 (*PfMAP2*) and describe for the first time its activity, function, and expression throughout the cycle of the main malaria parasite* Plasmodium falciparum*.

Degarege and Erko [9] summarize the findings of epidemiological studies of* Plasmodium* and helminth coinfection, emphasizing the impact of the coinfection on malaria in a review article.

Mbengue et al. [10] present the results of an IgG binding assay able to discriminate the outcome of cerebral malaria cases in Senegal, with the prospect of a potential functional-associated assay for symptomatic malaria analysis.

Finally, Ivanescu et al. [11] discuss the association between increasing rates of malaria over a time period where environmental temperature is also increasing. Based on an extrapolation of the climate conditions they predict the risk of malaria re-emergence in Romania.

*Aedes aegypti* is one of the most important disease vectors in the world. Its control is the main tool available to fight transmission of diseases such as dengue, Zika, or chikungunya. Bellinato et al. [12] bring us an overview of the resistance profile of* A. aegypti *to several insecticides in Brazil, the country most affected currently by dengue and Zika virus.

Presently, lice infestations occur worldwide despite great efforts to maintain high standards of public health. Infectious diseases transmitted by lice remain a public health concern in populations living in crowded and unsanitary conditions, a matter of great concern regarding refugee care. Sangaré et al. [13] detail a state-of-the-art review on the “Management and Treatment of Human Lice,” which, like for many other vectors, have been highly affected by insecticide resistance.

Leishmaniasis in the Old and New World is transmitted via the bite of phlebotomine sand flies and is caused by kinetoplastid parasites belonging to the genus* Leishmania*. This collective group of diseases is distributed in 88 countries around the globe with up to 1.6 million estimated cases per year [14–16]. To date, there is no human vaccine and treatment is largely based in 1940's antimony-based drugs which use causes distressing side effects. To date, control of transmission of leishmaniasis has focused mainly on the use of insecticides, a strategy that will eventually result in selection of resistant strains of insects, as seen in other insect vectors, such as* Aedes aegypti* and* Anopheles *spp. The World Health Organization has emphasized the need of developing novel strategies and research on these neglected vector-borne diseases. This special issue includes two papers on leishmaniasis.

Hijjawi et al. [17] assessed the use of molecular tools for the study of human leishmaniasis cases in Jordan, showing that identification of parasite species from dry samples is possible and improves clinical diagnosis. Besides that, they report the occurrence of* Leishmania tropica* in Jordan, which could be a new epidemiological concern related to the Syrian crisis.

Mohamed Mahmoud et al. [18] identified the presence of* Leishmania major* and* L. tropica* in a cutaneous leishmaniasis foci in Errachidia, Morocco, using molecular techniques. The authors also discuss the geographical distribution of* Leishmania *spp. in this area and report, for the first time, the presence of* L. tropica* in the region.

Chagas disease is still a major parasitic disease in the Americas, with up to 7 million cases worldwide. It has gained recent attention due to imported cases in nonendemic regions like USA (>300,000 cases) or Europe (>80,000 cases) [19]. It is caused by the parasite* Trypanosoma cruzi*, which is transmitted by the feces and urine of triatomine kissing bugs. The resurgence of vector populations and some reports of insecticide resistance in triatomines make of special interest the study and development of new strategies for control of these vectors [20]. Henriques et al. [21] studied in detail the action of the Insect Growth Regulator (IGR) Triflumuron (TFM) in* Rhodnius prolixus*, which is a model for triatomine biology. This work shows important effects of TFM on female fertility and gives some new insights in the mechanism of action of this insecticide, as impairments in diuresis and chitin turnover with reflections in insect immunity.

Taken together, the articles in this special issue cover several important aspects of major VBDs, highlighting not only their impact in human health, but also their significance for the development of new concepts and tools for the medical and biological research.

*Tribute to Dr. Bruce Alexander.* During our time as guest editors for this special number, we lost our friend and colleague John Bruce Alexander, who sadly passed away last March after a quiet battle against cancer. Bruce worked extensively in countries such as Colombia, Ecuador, and Brazil, being an international reference in the field of sampling and control of phlebotomine sandflies. Besides contributing with essential reviews and discussions to the field of leishmaniasis [22–25], he participated in seminal works related to the most diverse topics related to this neglected tropical disease, including transmission by midges in Australia [26], insecticide use and resistance in sandflies [27–30], role of chickens [31, 32], dogs [33, 34], and plants [35, 36] in the maintenance of vectors and parasite life cycle, taxonomic description of new sand fly species [37, 38], sand fly sex pheromones [39, 40], RNAi in sand flies [41], and biological control of sand flies [42]. His work relates to current and successful interventions for disease control, especially in the New World. Because his contribution to this special issue was interrupted in the presubmission stage, we decided to honor him with a very short obituary note here.

Bruce graduated in biology in the University of Edinburgh in 1979 and got his M.S. degree in entomology in the University of London. In his time there, Bruce met Dr. Robert Killick-Kendrick, who introduced him to the study of sand flies. A few years later, Bruce carried out a Ph.D. degree in Florida University on the ecology of sand flies in Northeast Colombia, learning Spanish and Portuguese and working on different aspects of phlebotomine biology. Later he moved to Brazil, where he worked at the Universidade Federal de Minas Gerais and FIOCRUZ. He moved back to the UK and in 2005 started working at the Liverpool School of Tropical Medicine. Later in his professional life, Bruce decided to become an independent researcher and together with his wife Cristina, he successfully started Xeroshield Ltd. in 2005 and Garrapat Ltd. in 2015. The spirit of both companies was to offer “practical solutions to insect control and vector-borne disease prevention to offer safe, sustainable, environmentally friendly alternatives to chemical pesticides.” He was an enthusiastic ornithologist, as well. He is survived by his wife, Cristina, and his son, Patrick. We will dearly miss his sense of humor, his wit, and passionate and argumentative discussions.
